# Facile and scalable preparation of superhydrophobic brass mesh for efficient and rapid separation of oil and water

**DOI:** 10.1038/s41598-024-63428-7

**Published:** 2024-06-04

**Authors:** Fatemeh Asjadi, Maliheh Yaghoobi

**Affiliations:** 1 Department of Materials Science and Engineering, Faculty of Engineering, University of Zanjan, P.O. Box 45371-38791, Zanjan, Iran; 2 Department of Chemical Engineering, Faculty of Engineering, University of Zanjan, P.O. Box 45371-38791, Zanjan, Iran

**Keywords:** Brass mesh, Superhydrophobic, Oil/water separation, Electrochemical etching process, Surface modification, Structural materials, Techniques and instrumentation, Chemical engineering

## Abstract

A facile method for preparing superhydrophobic brass mesh is proposed based on electrochemical etching and surface modification. The impact of processing time and the electric potential of the electrochemical etching were studied on the contact angle (CA) of the mesh. The samples were examined using scanning electron microscopy, Energy-dispersive X-ray spectroscopy analysis, X-ray diffraction, and Fourier-transform infrared spectroscopy. The electrochemical etching process caused the decrement of wires’ thickness and imposed roughness. Results showed more dissolution of zinc than copper under 3 V of the electric potential and the processing times of 3 and 6 min. The optimum condition of electrochemical etching was obtained under the electric voltage of 3 V for a processing time of 6 min, which led to a CA of 155.5 ± 3.2°. The thickness of the mesh wires decreased by 17.7% due to electrochemical etching in this sample. This sample also showed low adhesion for a water drop. The efficiency of oil/water separation was above 95 for the xylene and ethyl acetate in a batch system. The effect of the flow rate of the oil–water mixture on separation efficiency was also examined. The optimum flow rate was 0.8 ml s^−1^ with a high separation efficiency of 96.8% for xylene/oil separation.

## Introduction

Numerous industries, including petrochemical, textile, metallurgical, food, and catering, generate oily wastewater. The improper discharge of this oily wastewater impacts the environment and human health adversely^1^. Several methods are employed for oil and water separation, such as gravity settling^2^, centrifugation^3^, gas flotation^4^, adsorption^5^, and membrane filtration^6,7^. In addition, notable improvements in surface science, especially materials with specific wetting properties, have led to a new way for oil/water separation^8,9^. As a result, superhydrophobic and superhydrophilic surfaces were recently developed in the form of membranes^10^, adsorbent^11^, adsorptive fibers^12^, composites^13^, and mesh^14^ for the separation of water and oil.

Among various kinds of membranes, superhydrophobic membranes have gained significant attention. These highly water-repellent membranes can potentially improve the efficiency of water filtration systems, oil/water separation, and other applications^14^. The water droplets remain on the surface of the mesh while the oil passes through, resulting in efficient separation^15^.

There are several methods for producing superhydrophobic metal meshes. These methods usually possess surface roughening and modification steps. The former can be performed using electrochemical etching^16^, sputtering^17^, hydrothermal^18^, solvothermal^19^, electrodeposition^20^, and chemical vapor deposition^21^. The latter is commonly achieved using organic^22^ and inorganic^23^ substances. For instance, the modification of the porous stainless steel mesh with trichloro(octadecyl)silane through the dip coating method was adopted to improve its superhydrophobicity with a contact angle (CA) of 152°^24^. In addition, a CA of 165.8° was reported for superhydrophobic stainless steel mesh fabricated by applying long-chain silanes via the dip coating method^25^. Candle sooth coating followed by modifications was also used to make the stainless steel meshes superhydrophobic for oil/water separation^26^. Spraying superhydrophobic ZnO nanoparticles is another method used to form superhydrophobic stainless steel mesh leading to oil/water separation efficiency larger than 98.5%^27^. Ndong et al*.* used TiO_2_ coating to prepare superhydrophobic stainless steel meshes for oil/ water separation application^28^.

The superhydrophobic brass mesh was also fabricated and applied for oil/water separation. Deposition of layered double hydroxide followed by modification with stearic acid (SA) used for the preparation of superhydrophobic brass mesh with CA of 160° and high efficiency in separating various oils and water^29^. Laser ablation is also used as a one-step process for the hydrophobization of brass mesh. However, the as-lasered samples are superhydrophilic and they turn hydrophobic after aging over time^30^. The superhydrophobic brass meshes were also fabricated using pulsed electrodeposition of hydroxyapatite^31^ followed by surface modification.

Among the mentioned methods, electrochemical etching is a versatile process that allows for control over the pore size and shape in the metal meshes. It involves dissolving metal from a thin sheet using an electrolyte solution under an electric potential. This process can create a porous surface with micro/nano features that contribute to the water-repellent properties of the mesh. The resulting structure is highly durable and corrosion-resistant, making it ideal for outdoor applications^32^. Other properties, such as mechanical strength, thermal stability, and surface roughness, can also be controlled to meet specific needs^33^. Despite the facile and fast electrochemical etching process, few researchers have utilized it to fabricate superhydrophobic metal meshes. For example, Yu et al*.* used anodic oxidation of stainless steel mesh using voltage from 10 to 40 V to fabricate a superhydrophobic mesh. The separation efficiency of different oils was more than 95%^1^. Negri et al*.* used an electrochemical process using an inert electrolyte that contains stearic acid for the fabrication of superhydrophobic brass and bronze meshes. The stearate compounds with flower-like and nanotube morphology formed on the brass and bronze meshes while superhydrophobicity was achieved in the latter morphology^34^.

In this research, the brass mesh was roughened using the electrochemical etching process, and it was modified with SA. The effects of parameters such as electrochemical etching time and voltage on the morphology, surface composition, and superhydrophobic properties were investigated. The separation efficiency of oil and water was measured for the optimized sample. The effect of flow rate on the separation of oil/water was also studied.

## Materials and methods

### Fabrication of superhydrophobic brass mesh

A commercially available brass mesh was cleaned with acetone and deionized water to remove impurities. Then, a one molar Cu(SO_4_)_2_·5H_2_O, obtained from Kian Kaveh Azma, solution in deionized water was prepared. The brass mesh and pure copper foil were used as anode and cathode, respectively. The brass mesh was set between two copper foils to create equal conditions on both sides of the mesh. The distance between the cathode and anode was adjusted to 2 cm. Three sets of experiments were performed under electric potentials of 2, 3, and 4 V. Electrodes were connected to a direct current (DC) power supply. The current flowed for a fixed duration of 3, 6, and 9 min. After cutting off the electric potential, the samples were submerged in water to cease any further reactions. The experiments were repeated using 0.5 M Cu(SO_4_)_2_·5H_2_O solution. The electrochemically etched meshes were immersed in 0.1 molar SA solution in 99.6 wt% ethanol for 10 min at room temperature, in the modification step. Subsequently, the samples were thoroughly washed with the same grade of ethanol for half a minute and dried with a laboratory dryer. The samples were named as follows: Cu (the solution concentration)-electrochemical etch voltage-duration of electrochemical etching. For instance, Cu(1)–2–3 refers to the sample electrochemically etched with one molar Cu(SO_4_)_2_·5H_2_O solution with an electric potential of 2 V for 3 min.

### Characterization of the superhydrophobic meshes

The morphology and elemental analysis of the brass meshes were investigated using scanning electron microscopy (MIRA3TESCAN SEM) equipped with EDS analysis. The thickness of the wires and any other necessary measurements on the SEM images were recorded using ImageJ software^35^. The Fourier-transform infrared spectroscopy (FTIR) spectroscopy was conducted using a NICOLET iS10 (Thermo Scientific, USA) spectrometer with an attenuated total reflectance (ATR) accessory. A homemade contact angle goniometer consisting of a USBFHD01M color industrial camera attached to a lens with a variable magnification from 10x–45x was employed for measuring the static contact angle through the sessile drop technique. A deionized water droplet in a volume of 6 μl with total dissolved solids of ≤ 0.02 ppm was used for CA measurements. The droplet shape analysis was conducted by B-spline snakes using Image-J software^36^. The average CA and its standard deviation were obtained with at least four trials.

The mechanical stability of the samples was tested using abrasive and tape-peeling tests. The prepared superhydrophobic mesh was moved repeatedly on the SiC P400 sandpaper under the 100 g weight in an abrasive test. The movement distance was 10 cm and the test was performed for 40 cycles. The CA was measured every ten cycles. The superhydrophobic sample was pressed and peeled off using the tape for the peeling test. The CA was measured every ten cycles. The chemical stability of the superhydrophobic mesh was studied by investigation of CA after immersion of the sample at 1 and 0.1 M solution of HCl and NaOH for 2 h and 24 h.

### Oil/water separation

A frame-like boat was made from the optimum mesh and set on a beaker. The mixture of oil sample and water was poured onto the boat. To distinguish oil and water, methylene blue and turmeric were used to dye water and xylene, respectively. The excess turmeric was filtered before use. Xylene, ethyl acetate, and edible oil were used as sample oils. The oil/water separation efficiency and reusability of the mesh were tested for three oils. The volume of oil before and after the process was measured and used to determine oil/water separation efficiency ($\eta$) according to the following formula:

1 $$\eta \left( \% \right) = \frac{{V_{{\text{C}}} }}{{V_{0} }} \times 100\%$$

*V*_C_ represents the oil volume of collected oil after the separation and *V*_0_ is the oil volume in the original oil/water mixture.

Moreover, another setup was designed for continuous oil/water separation based on the study of Wang et al*.*^37^. In brief, the mixture of xylene and water was conducted into a pipe, which 3 cm of its length was substituted by superhydrophobic mesh. Water passes on the mesh without leakage, while the xylene penetrates through the mesh. The flow rate was varied to 0.1, 0.8, and 2.4 ml s^−1^. A 100 ml mixture of water and xylene with the same volume ratio was used for each experiment.

## Results and discussions

### Microstructure and surface characterization

**Figure 1:**
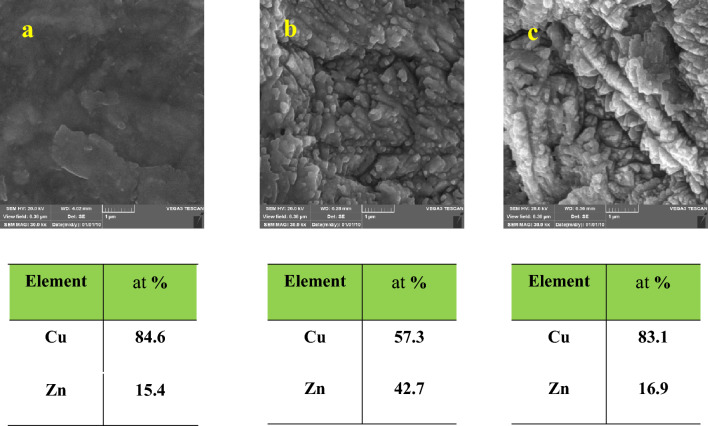
SEM images and EDS results of (**a**) initial mesh, (**b**) Cu(1)–2–6, (**c**) Cu(1)–3–6.

Microstructures and EDS elemental analysis of initial mesh and samples electrochemical etched for 6 min under different voltages are presented in Fig. 1. The initial mesh shows an entirely smooth surface. The average atomic percents of zinc and copper are about 84.6 at% and 15.4 at% on the surface of this sample, respectively. Since the mesh is not red brass, the composition shows the depletion of zinc from the surface of the initial mesh. A rough surface with 57.3 at% copper and 42.7 at% zinc is observed by electrochemical etching under an electric potential of 2 V for 6 min. The percentage of zinc and copper decreases and increases by raising the voltages of the electrochemical etching process to 3 V, respectively. Decrement in zinc percentage is acceptable considering the lower electrochemical potential of the zinc. The Zinc converts to Zn^2+^ according to the following reaction with a standard electrochemical potential of 0.76 V:

2 $${\text{Zn}} \to {\text{Zn}}^{{{2} + }} + {\text{2e}}^{ - }$$

Copper releases electrons under electric potential with one of the following reactions:

3 $${\text{Cu}} \to {\text{Cu}}^{{{2} + }} + {\text{2e}}^{ - }$$

4 $${\text{Cu}} \to {\text{Cu}}^{ + } + {\text{e}}^{ - }$$

The standard potential of reactions 3 and 4 are − 0.34 V and − 0.52 V, respectively. Therefore, zinc is more prone to electrochemical dissolution than copper. Selective dissolution in electrochemical etching under 3 V electric potential increases the roughness.

**Figure 2:**
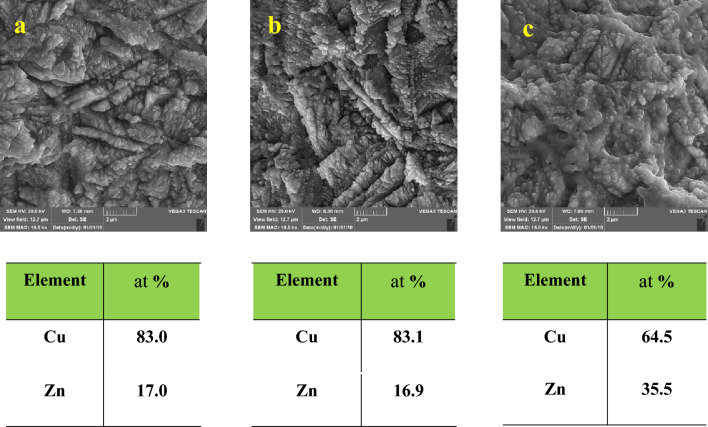
SEM images and EDS results of (**a**) Cu(1)–3–3, (**b**) Cu(1)–3–6, (**c**) Cu(1)–3–9.

Figure 2 revealed the microstructures and EDS results of the samples electrochemical etched under voltage 3 V with various processing times. Electrochemical etching for 3 and 6 min leads to an increment of copper percentage, as explained before. There is no difference between the composition of these two samples. However, the notches on the surface of the samples with 6 min of electrochemical etching are deeper than those with 3 min. The roughness decreased after 9 min of electrochemical etching, and the zinc and copper percentages are 35.5 at% and 64.5 at%, respectively. The copper enters the electrolyte over time, which could be due to the depletion of the surface from zinc. Therefore, the preferred oxidation of zinc has not occurred. The topography changed, and the surface became smoother due to a lack of preferred dissolution. As mentioned before, this sample dissolved in edges, and the remaining part was delicate. It was practically unusable since it was too flexible and thin.

**Figure 3:**
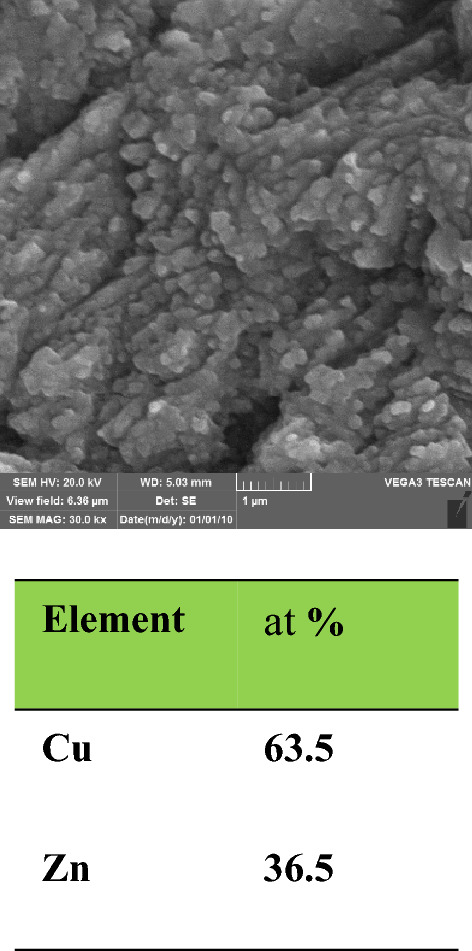
SEM images of the sample Cu(0.5)–3–6.

The morphology and composition of the sample Cu(0.5)–3–6, Fig. 3, looks like the sample Cu(1)–2–6. Therefore, decreasing the voltage of the process and the electrolyte concentration have the same impact on the morphology and composition of the samples. This conclusion is sensible since both of these factors decrease the electric current. The CAs of these two samples are also similar which will be discussed in “CA measurements” section. It is noteworthy that the surface morphology and composition of these samples were also similar to sample Cu(1)–3–9.

In conclusion, in a small and large amount of etching, the composition of etched samples was close. In addition, low roughness appeared on the surface in this condition. The former was due to low dissolution, and the latter was for harsh dissolution. The surface layer was removed at the low electric potential and low electrolyte concentration. However, neither zinc nor copper dissolved significantly at these conditions, for example, samples Cu(1)–2–6 and Cu(0.5)–3–6. In addition, both of them dissolved in high electric potential and decent etching time, for instance, sample Cu(1)–3–9. Both cases led to less roughness of the surface. Selective dissolution of zinc and, consequently, more roughness of the surface occurs at a medium degree of etching. These cases are the optimum conditions for superhydrophobic mesh preparation. In this study, the most selective dissolution of zinc, based on EDS analysis, and the most roughening, based on SEM images, occurred in the sample Cu(1)–3–6.

**Figure 4:**
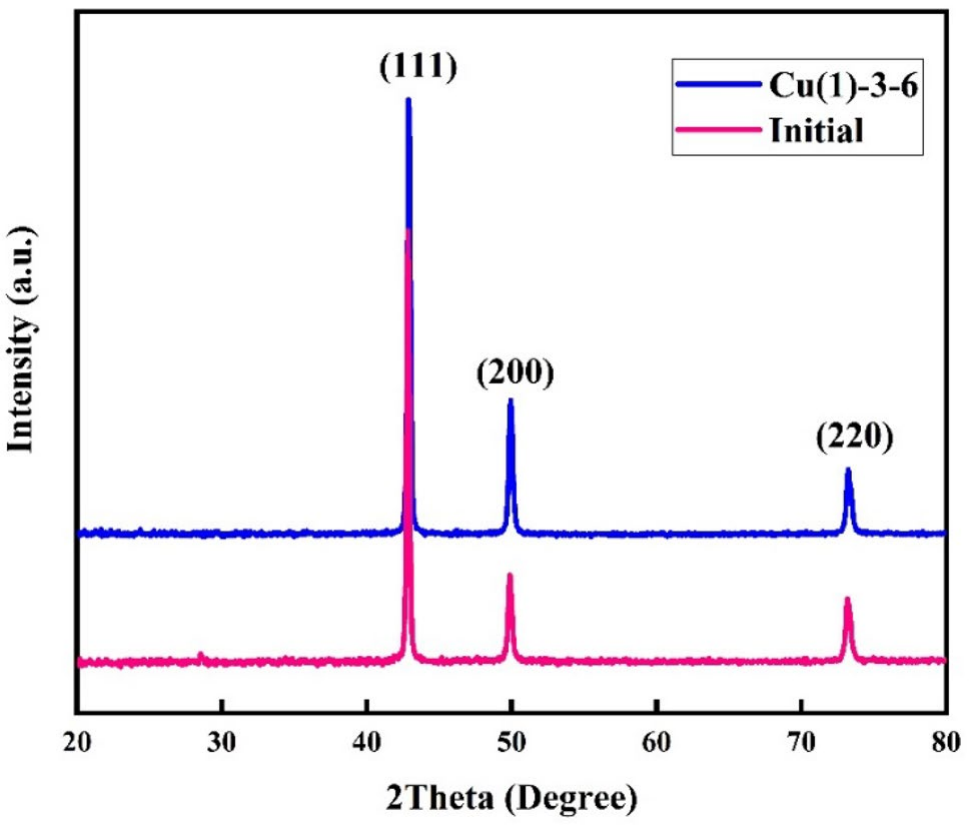
XRD patterns of the initial mesh and Cu(1)–3–6 sample.

XRD patterns of the sample Cu(1)–3–6 and initial mesh are shown in Fig. 4. The observed peaks at ~ 42.5°, ~ 49.78°, and ~ 73.35° attributed to planes (111), (200), and (220) of fcc α-brass according to JCPDS NO.50–1333, respectively. The (200) peak shifted to a higher 2θ in Cu(1)–3–6 sample compared to the initial mesh. Phase α-brass can be considered a substitutional copper alloy. The atomic radius of zinc, 1.34 A, is larger than copper, 1.28 A^38^. The EDS analysis shows the preferred dissolution of zinc from the structure. Therefore, depleting the zinc from the structure of brass would decrease the plane distance and shift the XRD peaks to a larger 2Ɵ. In addition, the intensity of this peak also increased in sample Cu(1)–3–6. This is probably due to more dissolution of other planes than the (200) plane. This led to the preferred orientation on the (200) peak and its intensification. Therefore, the notches in SEM images of this sample may be attributed to the preferred orientation phenomenon.

**Figure 5:**
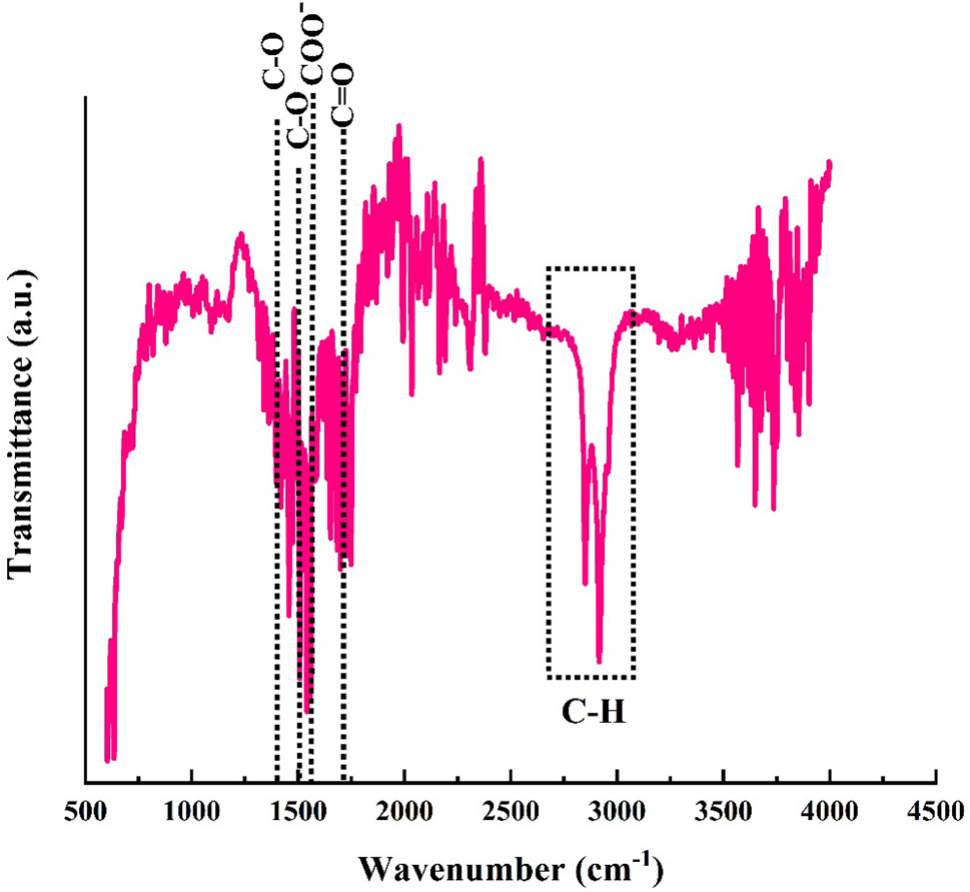
FTIR Spectrum of SA-modified superhydrophobic sample.

Figure 5 demonstrates the FTIR spectrum of the SA-modified superhydrophobic mesh. The absorbance band of pure SA was well-studied before. The peaks in the range of 730 to ~ 1500 are observed in the SA FTIR spectrum^39,40^. For instance, the band at 1430 cm^−1^ is due to the presence of C–O^41^, The low-intense peaks in the range of 1190–1350 cm^−1^ are attributed to the carboxylic acid group of SA^41^. In addition, two peaks of SA at 2848 and 2919 cm^−1^ arises from the C–H symmetric and asymmetric stretching vibrations^41^. FTIR spectrum of mesh possesses the main absorbance band of SA. These peaks are described grafting of SA on the surface of the mesh. On the other hand, the occurrence of various peaks in the range of 1500–1750 cm^−1^ can be due to the absorption of groups from the air atmosphere and the COO group of SA reacting with copper and zinc in the brass mesh. The other research also reports the presence of peaks in the same range after surface modification with SA.^40,41^.

### CA measurements

**Figure 6:**
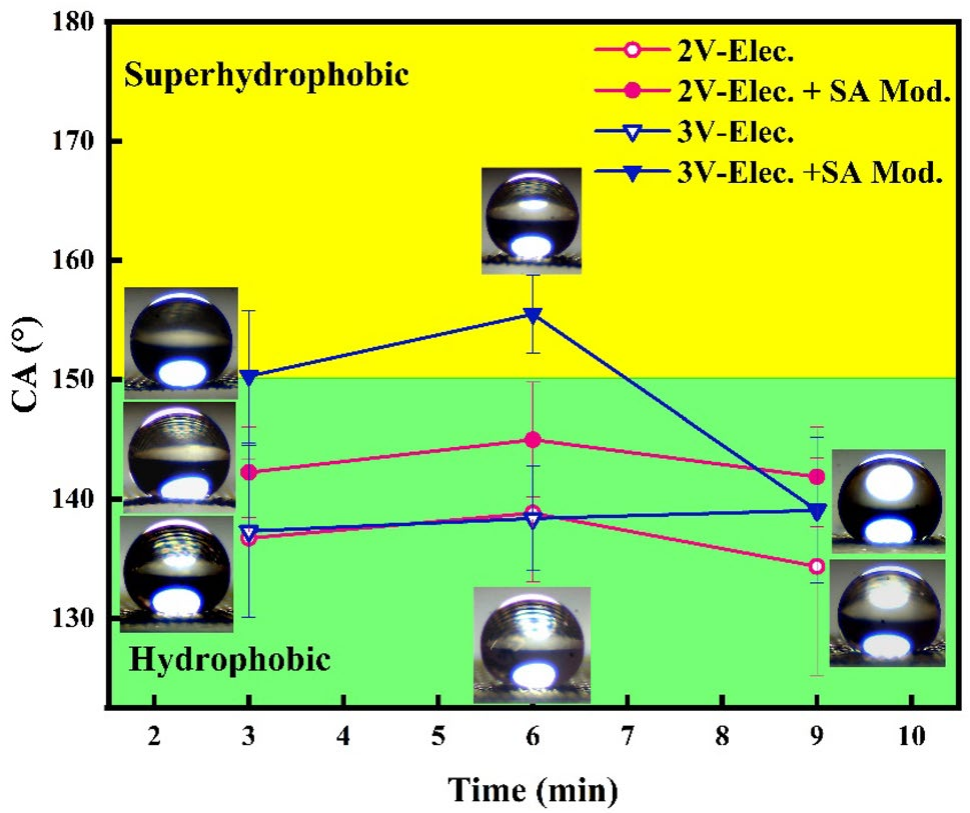
The CA of the samples electroetched in 1 M Cu(SO4)_2_·5H_2_O solution with various electroetching times before and after the SA modification.

Figure 6 exhibits the CA of the electrochemically etched samples under electric potentials of 2 and 3 V with different processing times before and after surface modification with SA. The Meshes were too etched and finally disappeared by increasing the electric potential to 4 V. The highly delicate part of the mesh in the circle shape remained in the center of it after 3 min and disappeared entirely in about 4 min. Therefore, this figure does not include the results of this voltage. Two samples, Cu(1)–3–3 and Cu(1)–3–6, were superhydrophobic, having a CA of more than 150°. The sample Cu(1)–3–6 showed the largest CA. The CA decreased by increasing the processing time to 9 min under an electric potential of 3 V. This trend was different in 2 V, and the difference between the CA of samples with different electrochemical etching times was negligible in this case. Surface modification with SA increases the CA of the samples except for Cu–3–9, in which the CA has not changed after surface treatment. This sample dissolved in most areas except the middle part, which became very thin.

**Figure 7:**
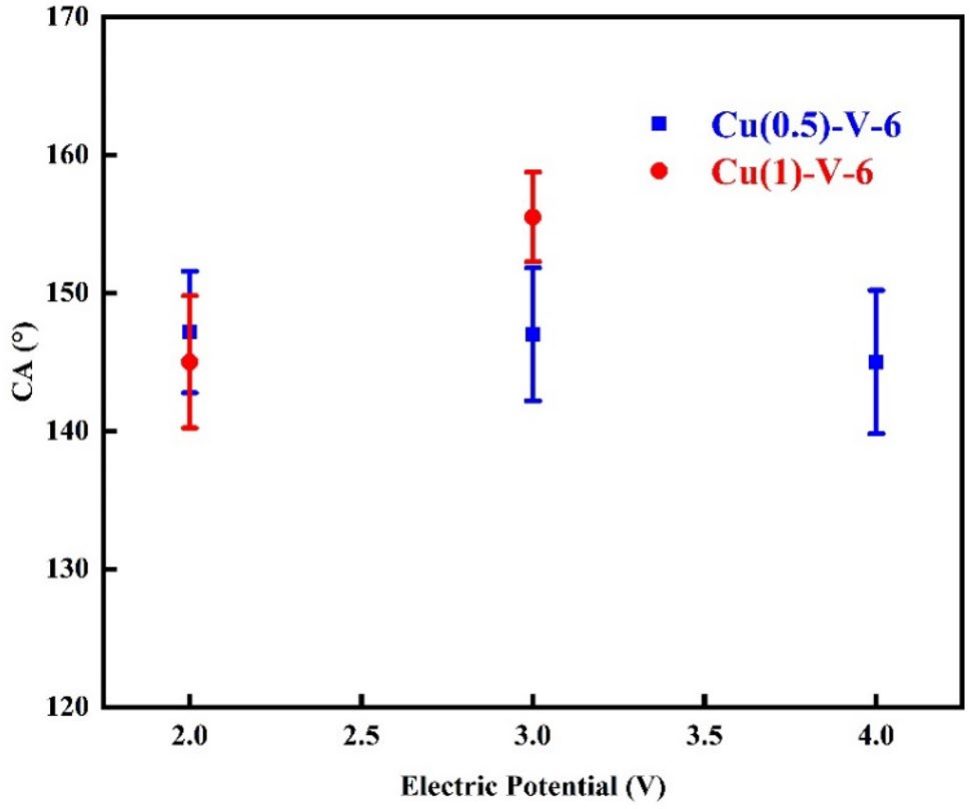
CA of the samples electrochemical etched in 0.5 and 1 molar concentrations electrolyte for 6 min in various potentials after SA modification.

Figure 7 shows the CA of the electrochemically etched samples using 0.5 M electrolyte for 6 min under different electric potentials after modification with SA compared to the result of one molar electrolyte. There is no meaningful difference between the CA of the samples electrochemical etched under 2 V electric potential in 0.5 and 1 M electrolytes. This is due to negligible etching for both samples in this voltage. The sample electrochemically etched in one molar electrolyte shows a larger CA than one treated in 0.5 M as the electric potential raised to 3 V. The resistance of the electrolyte solution increases, and the current decreases with decreasing the ions concentration. Lower current makes the lower charging particles according to the q = It relation. As a result, less etching occurs in a lower concentration. Therefore, increasing the electrolyte concentration causes more etching. However, there is a limitation in increasing the concentration. In higher concentrations, mesh etches either severely or entirely. The former leads to losing the mechanical strength, and the latter destroys the mesh. Moreover, increasing the etching decreases the thickness of the wires, which may be disadvantageous to superhydrophobic behavior.

In brief, the electrochemically etched mesh modified with SA exhibited the largest CA under 3 V electric potential for a processing time of 6 min. The initial mesh was hydrophilic. Therefore, the CA measurement was impossible since the drop was not set on it. The CA of SA-modified mesh, without electrochemical etching, was 134.2 ± 5.4°. It concludes that electrochemical etching and SA modification both play a critical role in the enhancement of the hydrophobicity of the mesh. The chemical composition and physical structure of the mesh can play a role in the superhydrophobic behavior of the mesh surface.

Based on the characterization results in “Microstructure and surface characterization” section and the CA of the samples, one can conclude that the electrochemical etching process roughened the surface of the mesh. In voltage of 3 V and duration of 6 min, the preferred dissolution of zinc occurred leading to more roughness. Subsequently, SA grafts and interacts with the mesh surface and decreases its surface energy, leading to an increment of CA.

### Investigation of wires’ thickness

**Figure 8:**
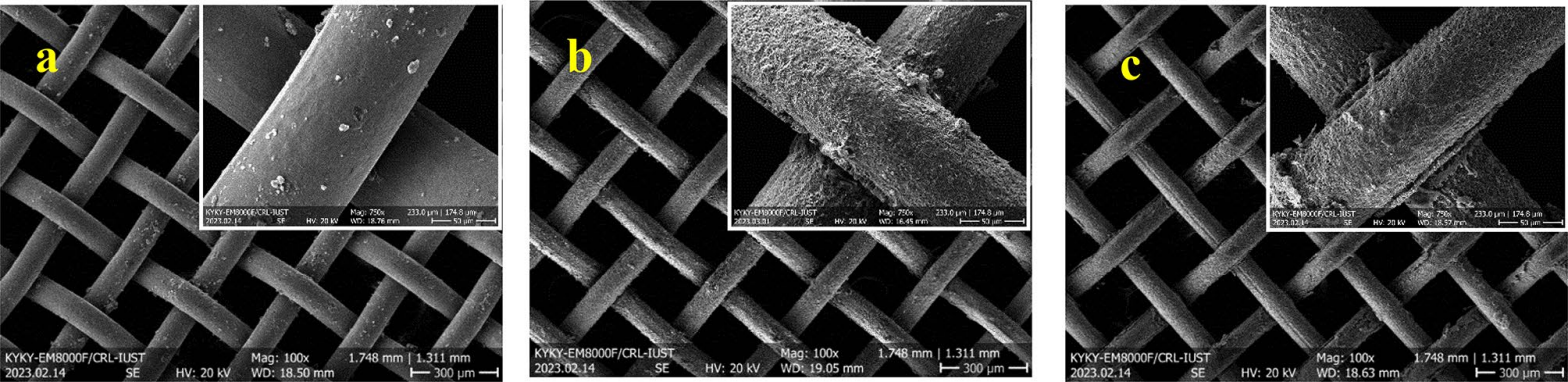
SEM images and EDS results of (**a**) initial mesh, (**b**) Cu(1)–2–6, (**c**) Cu(1)–3–6.

The SEM images, with 100 and 750 magnifications, of the samples electrochemical etched for 6 min and under various electric potentials accompanied by untreated brass mesh as a control sample are shown in Fig. 8. The images show the decrement in the thickness of the mesh’s wire due to the electrochemical etching. Decreasing the wire thickness increases the size of the openings.

**Figure 9:**
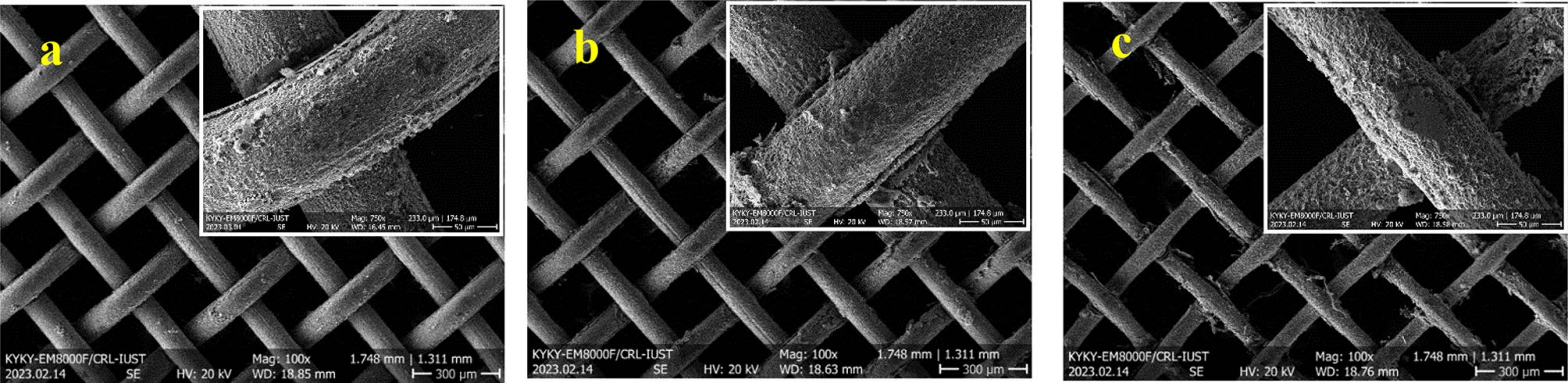
SEM images and EDS results of (**a**) Cu(1)–3–3, (**b**) Cu(1)–3–6, (**c**) Cu(1)–3–9.

SEM images of the samples with various electrochemical etching times under 3 V electric potential are shown in Fig. 9. The thickness of the wires decreased by increasing the treating time of the electrochemical etching.

Electrochemical etching changed the roughness as well as the thickness of the wires. The thickness of the wires of different samples is tabulated in Table 1. Droplet weight applied pressure on wires. Downward pressure increases by decreasing the wire thickness. The higher pressure causes the water to penetrate more in the roughness, and the dominance of the Wenzel model is more probable than Cassie-Baxter. Therefore, decreasing the CA by decreasing the wire thickness was expected. However, the results showed no meaningful relationship between the thickness and the CA. It means that roughness plays a critical role in CA determining since the roughness is not the same for all samples.

**Table 1:** thickness of wires and CA after electrochemical etched and SA modification.

Sample	Thickness (μm)	CA (degree)	L (μm)	$-\frac{cos\theta }{L}=\frac{\Delta P}{4\gamma }$
Unetched-SA	97.15 ± 2.75	134.2 ± 5.4	242.33	2.87 × 10^3^
Cu(1)–3–3	88.2 ± 2.2	150.3 ± 5.5	251.87	3.39 × 10^3^
Cu(1)-3–6	79.9 ± 2.9	155.5 ± 3.2	269.55	3.41 × 10^3^
Cu(1)–3–9	68.5 ± 3.5	139.1 ± 6.1	282.84	2.67 × 10^3^
Cu(1)–2–6	87.2 ± 2.9	145.0 ± 4.8	256.40	3.19 × 10^3^
Cu(0.5)–3–6	88.2 ± 3.0	147.0 ± 4.6	254.98	3.29 × 10^3^

In addition, the opening diameter increases by decreasing the thickness of the wire. Openings can be considered channels through which, the liquid penetrates, like capillaries. The theoretical intrusion pressure (ΔP) can be determined using the following eqution^20,42^:

5 $$\Delta P = \frac{2\gamma }{R}$$

6 $$= - \frac{\gamma Lcos\theta }{A}$$

7 $$= - \frac{4\gamma cos\theta }{L}$$

**Figure 10:**
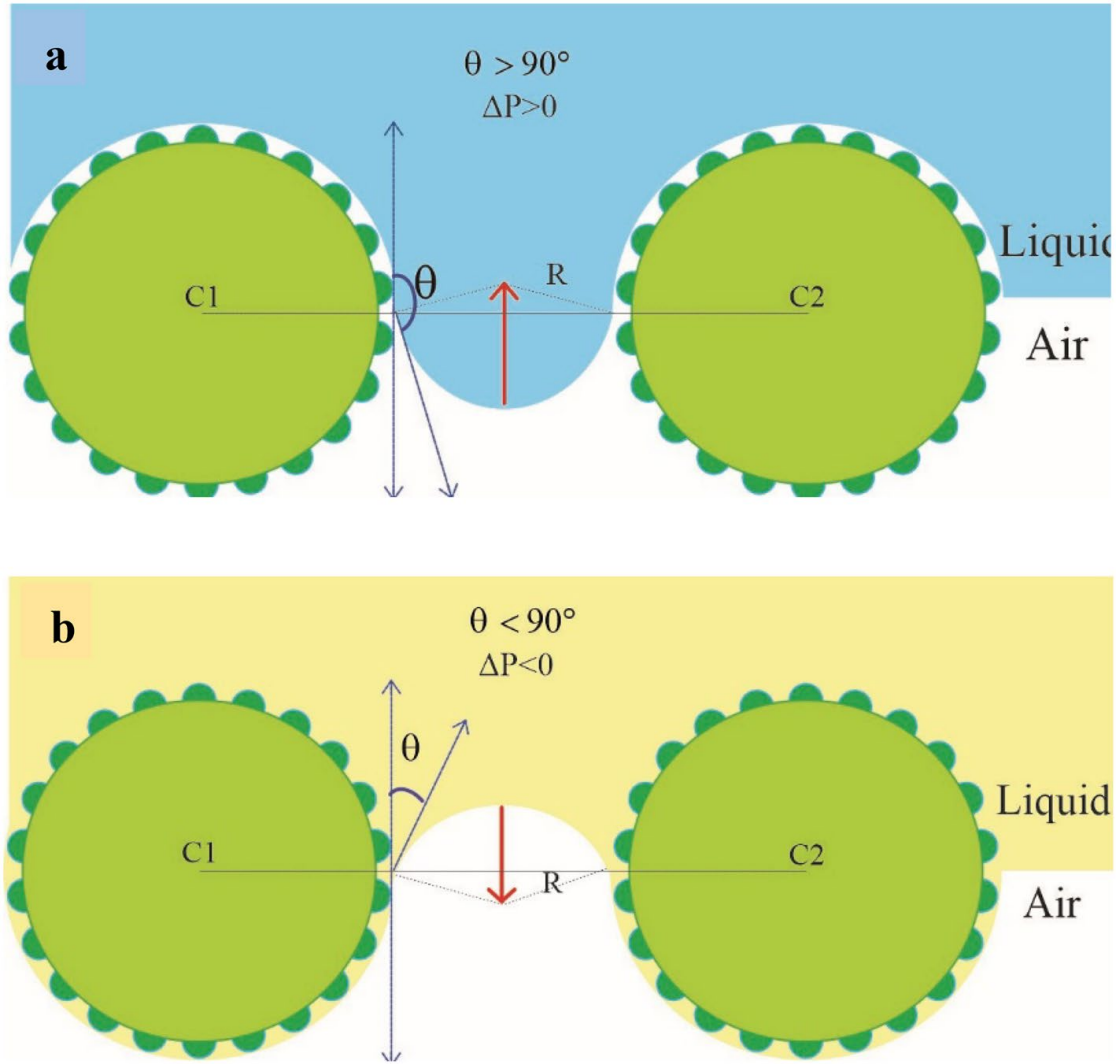
(**a**) The Superhydrophobic mesh with positive ΔP prevents water diffusion through the openings, and (**b**) The superoleophilic mesh with negative ΔP allows oil diffusion.

In which the $\gamma$ is surface tension, $\theta$ is the CA of the liquid on the solid, A is the pore area, L is pore diameter, and R is the radius of curvature. For hydrophobic surfaces, with the $\theta$ larger than 90°, the ΔP is positive and vice versa, Fig. 10. The $\frac{cos\theta }{L}$, which is proportional to $\frac{\Delta P}{4\gamma }$ was calculated in Table 1. The diameter of the square was considered as pore diameter. The highest $\frac{\Delta P}{4\gamma }$ is for sample Cu(1)–3–6, and the lowest is for Cu(1)–3–9. These results implied that the Cu(1)–3–6 sample with the largest CA and ΔP, assuming the equal γ for all samples, is the optimized sample for oil/water separation application.

### Adhesion test

**Figure 11:**
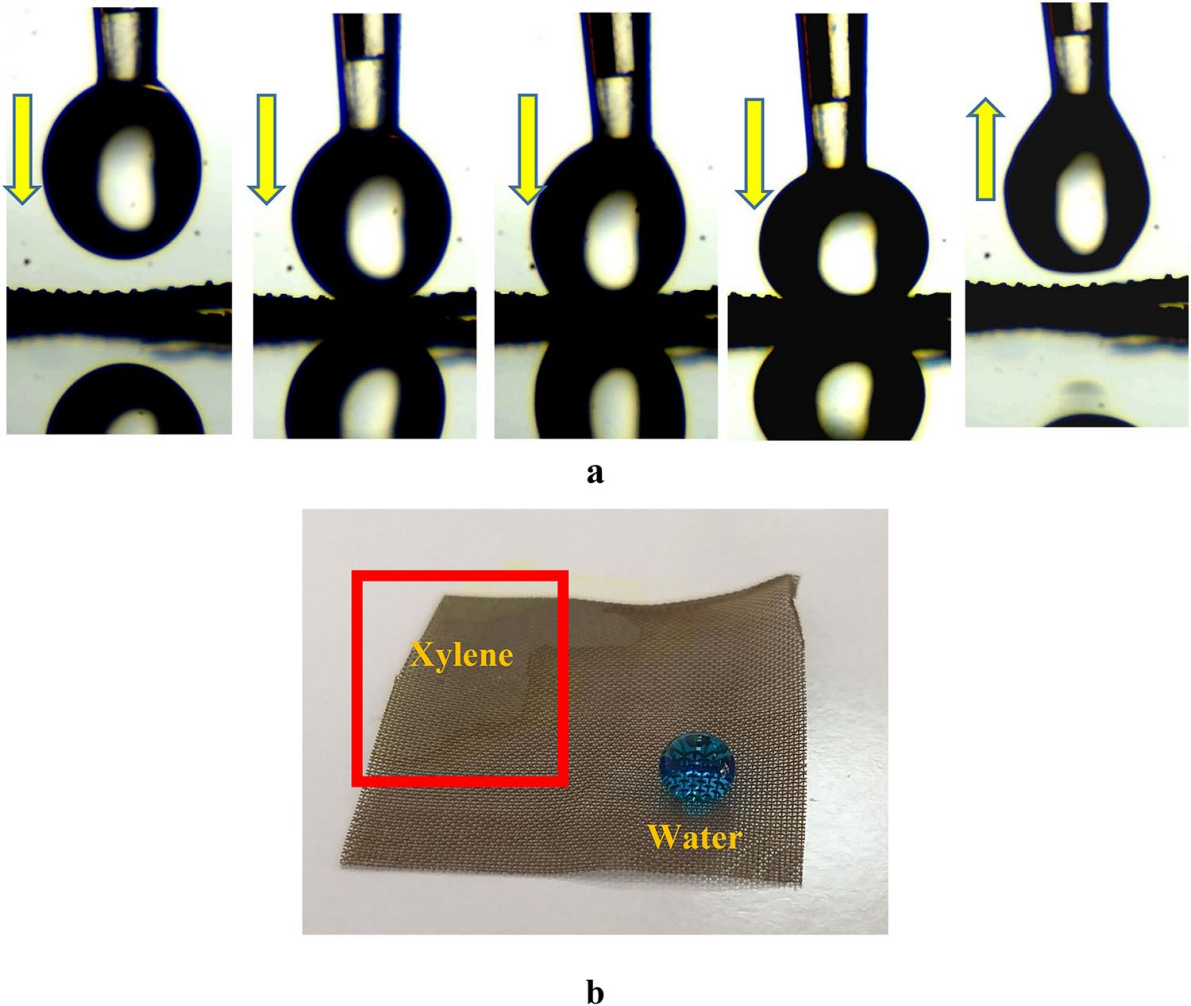
(**a**) Low adhesion of the sample, (**b**) 20 μL xylene and water drops on the sample Cu(1)–3–6.

The sample Cu(1)–3–6, possessing the largest CA and intrusion pressure, was selected for the adhesion test. No water remained on the surface when the droplet was taken downward, pressed against its surface, and dragged upward, as shown in Fig. 11a. On the other hand, it also has superoleophilic properties, as shown in Fig. 11b. The low water adhesion combined with the superoleophilic properties of the sample Cu(1)–3–6 confirms its applicability for oil/water separation.

### Mechanical and chemical stability

**Figure 12:**
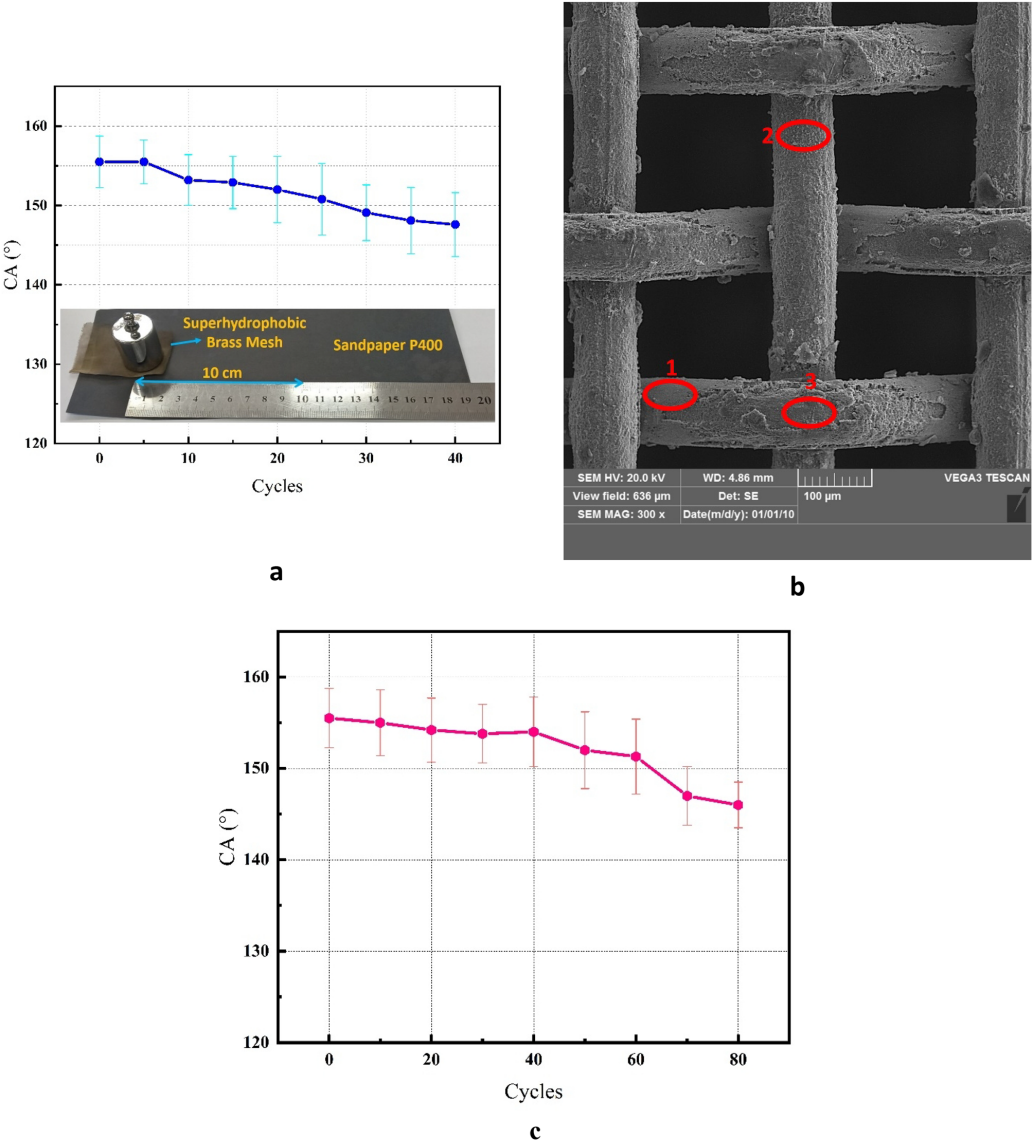
(**a**) The CA of the superhydrophobic mesh after various cycles of the sandpaper abrasion test, (**b**) SEM image of the mesh after abrasion test, (**c**) The CA of the superhydrophobic mesh after various cycles of the tape peeling test.

The mechanical stability and robustness of superhydrophobic meshes is an effective factor in practical applications. Figure 12a presents the result of the abrasion test. The CA decreases gradually as abrasion cycles increase. The mesh was still superhydrophobic, with CA above 150°, after 40 cycles. The SEM image of the mesh after the abrasion test is shown in Fig. 12b. The image revealed three regions including the un-electrochemically etched area around the intersection of wires, the intact electrochemically etched parts, and the damaged electrochemically etched regions due to abrasion, marked as 1, 2, and 3 in the image, respectively. Therefore, the CA decrement is due to the detachment of superhydrophobic film from the surface of the sample in some parts of the surface.

The effect of tape peeling on the CA of superhydrophobic mesh is shown in Fig. 12c. The CA showed a decreasing trend by increasing the peeling cycles. The mesh maintains its superhydrophobicity for 60 cycles. The results confirm the high mechanical stability of the superhydrophobic mesh.

**Figure 13:**
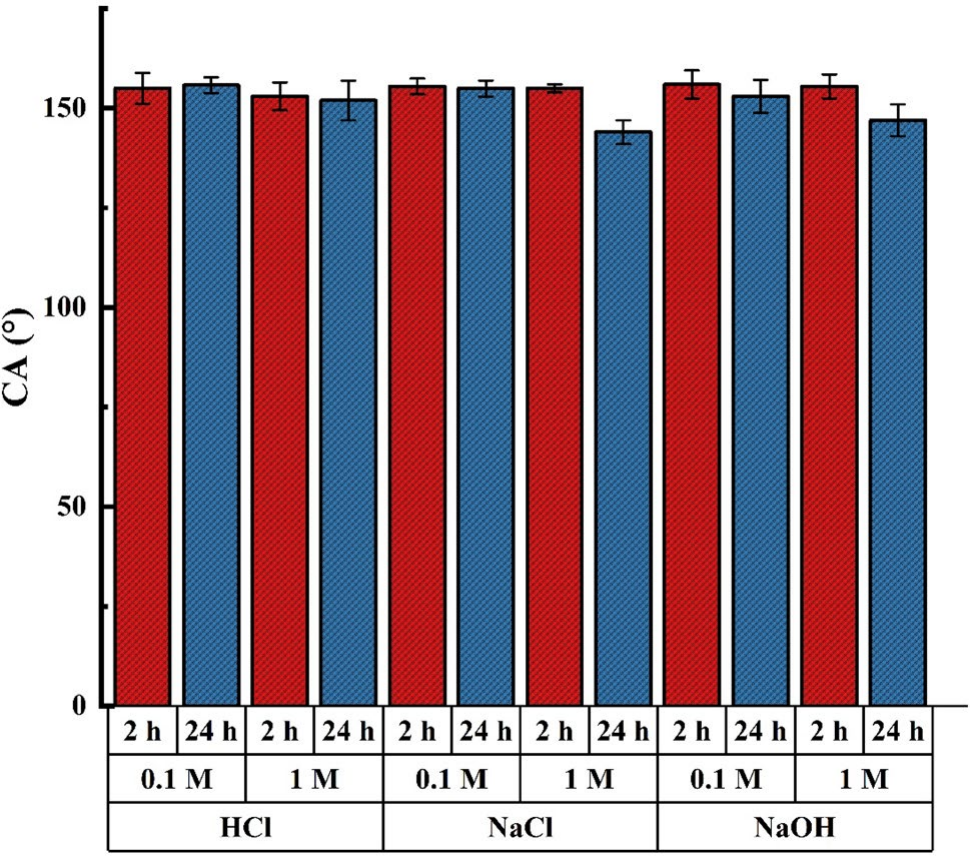
CA of the superhydrophobic brass mesh after immersion in various solutions for 2 and 24 h.

Figure 13 shows the CA of the superhydrophobic sample after immersion in 0.1 M and 1 M acidic, NaCl salt, and alkaline solution for 2 and 24 h. The CA showed a negligible reduction in 0.1 M concentration of all solutions even after 24 h. It is approximately constant after 2 h but decreases after 24 h in 1 M concentration of solutions. Moreover, it can be concluded that the sample is most sensitive to NaCl solution. In addition, the reduction of CA is larger in an alkaline solution than in an acidic one. In brief, the results confirm the chemical stability of the sample.

### Oil/water separation

**Figure 14:**
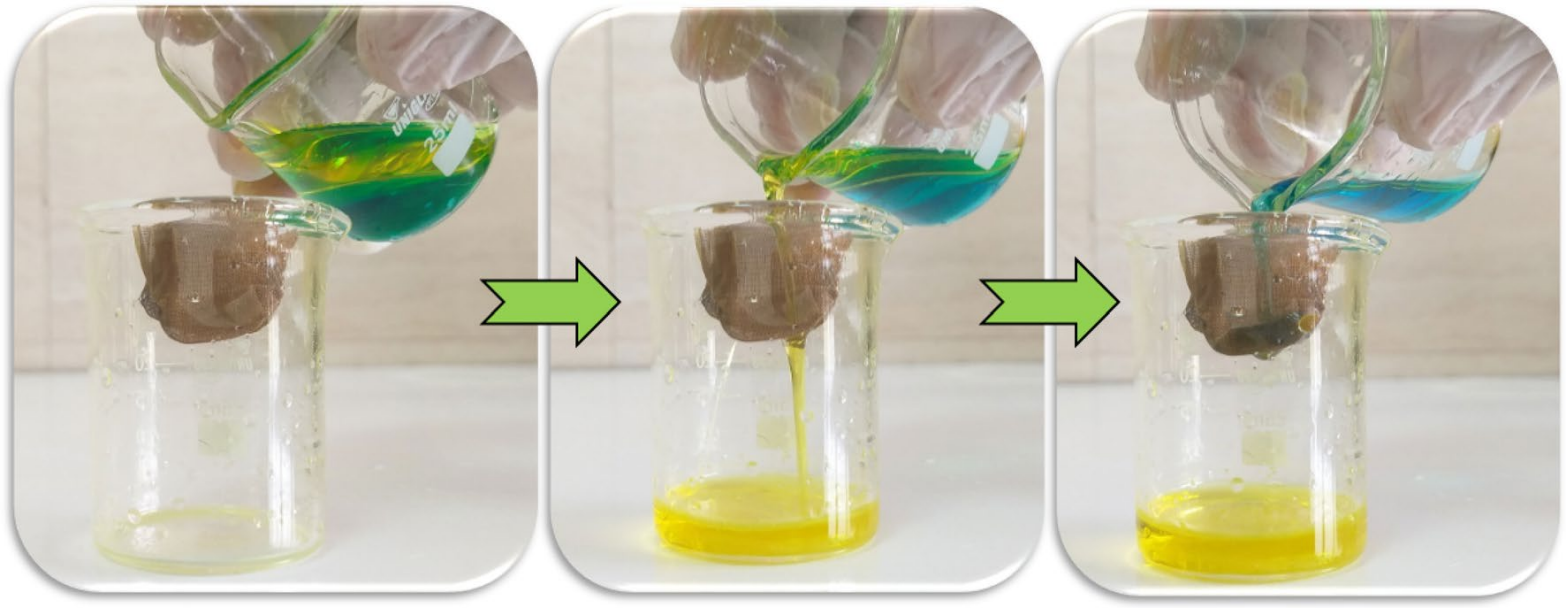
Experimental setup for calculating the efficiency of the oil/water separation.

**Figure 15:**
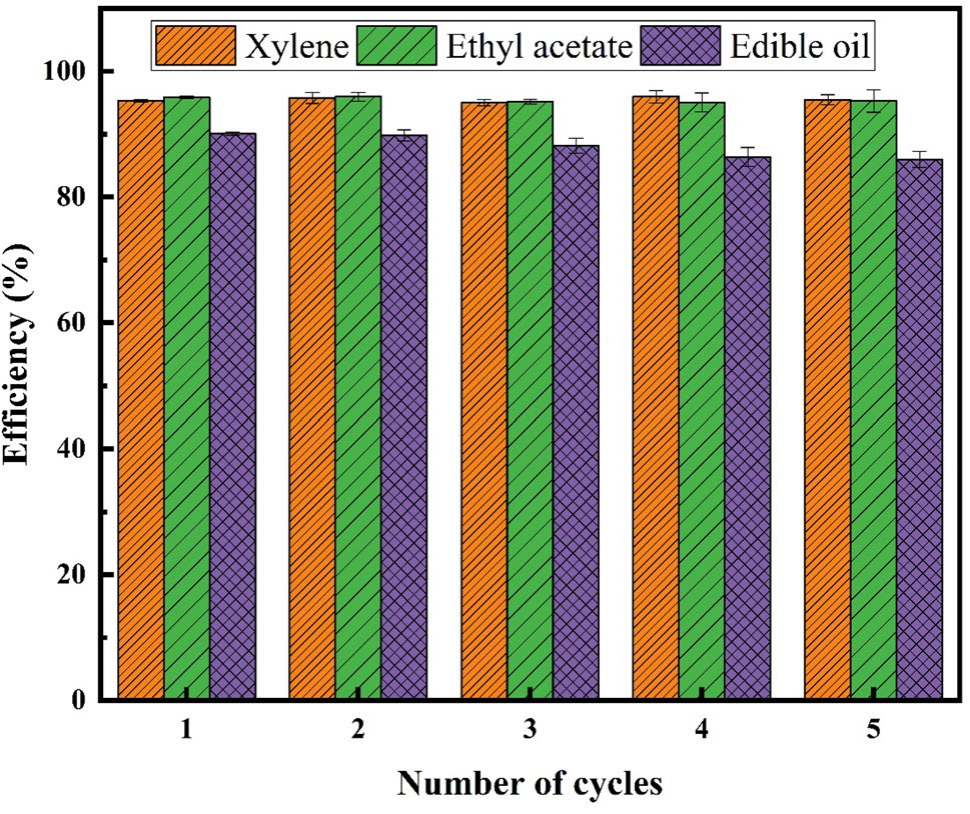
The efficiency and reusability of the meshes in oil/water separation for xylene, ethyl acetate, and edible oil.

Figure 14 shows the experimental setup of oil/water separation and the excellent performance of sample Cu(1)–3–6. Figure 15 represents the efficiency and reusability of the mesh in water/oil separation for three types of oil. The separation efficiency is higher for xylene and ethyl acetate compared to edible oil. Moreover, the separation efficiency was almost unchanged after 5 cycles for all three oils.

**Figure 16:**
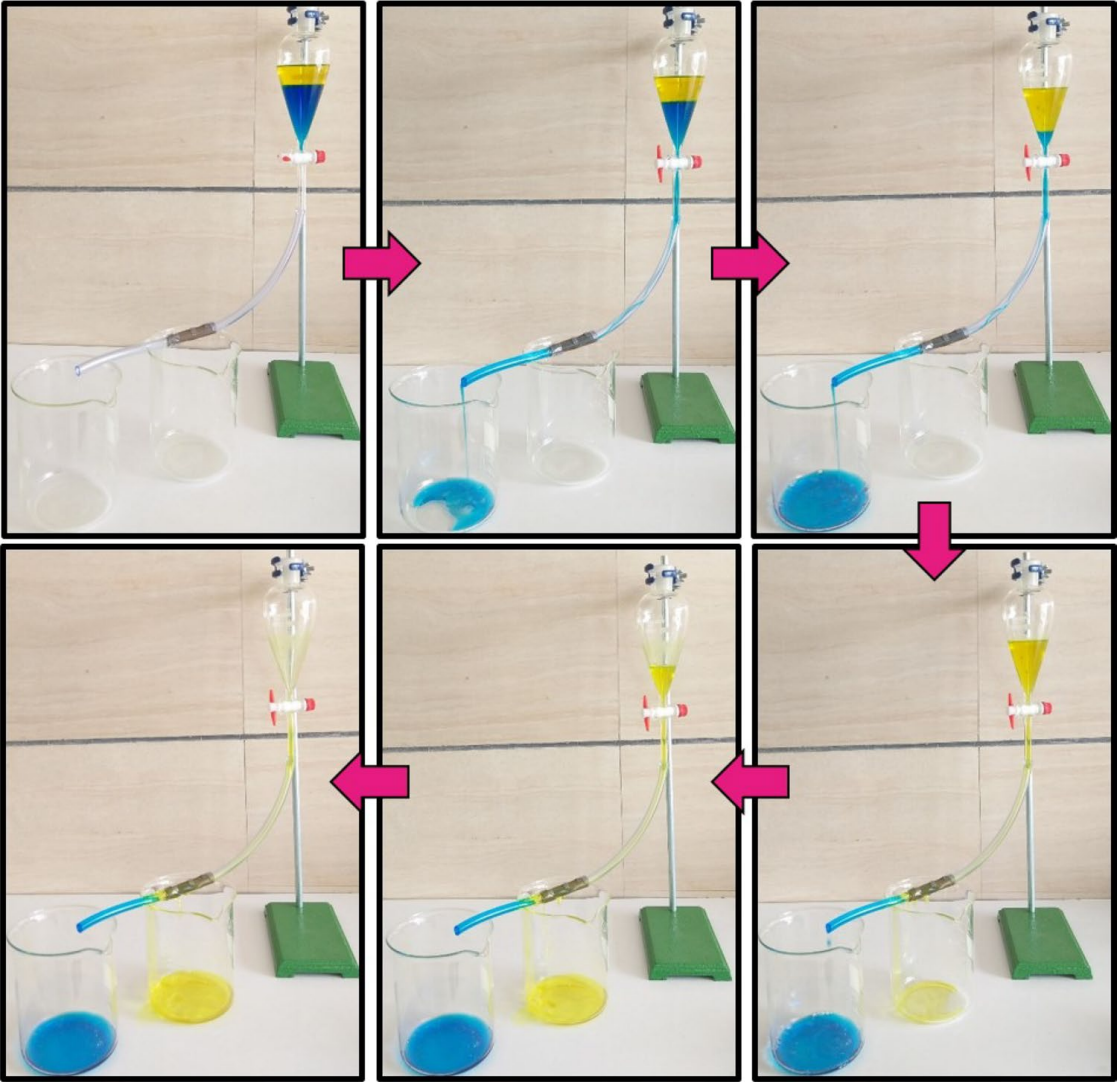
Experimental setup of oil/water separation and the performance of superhydrophobic mesh.

**Figure 17:**
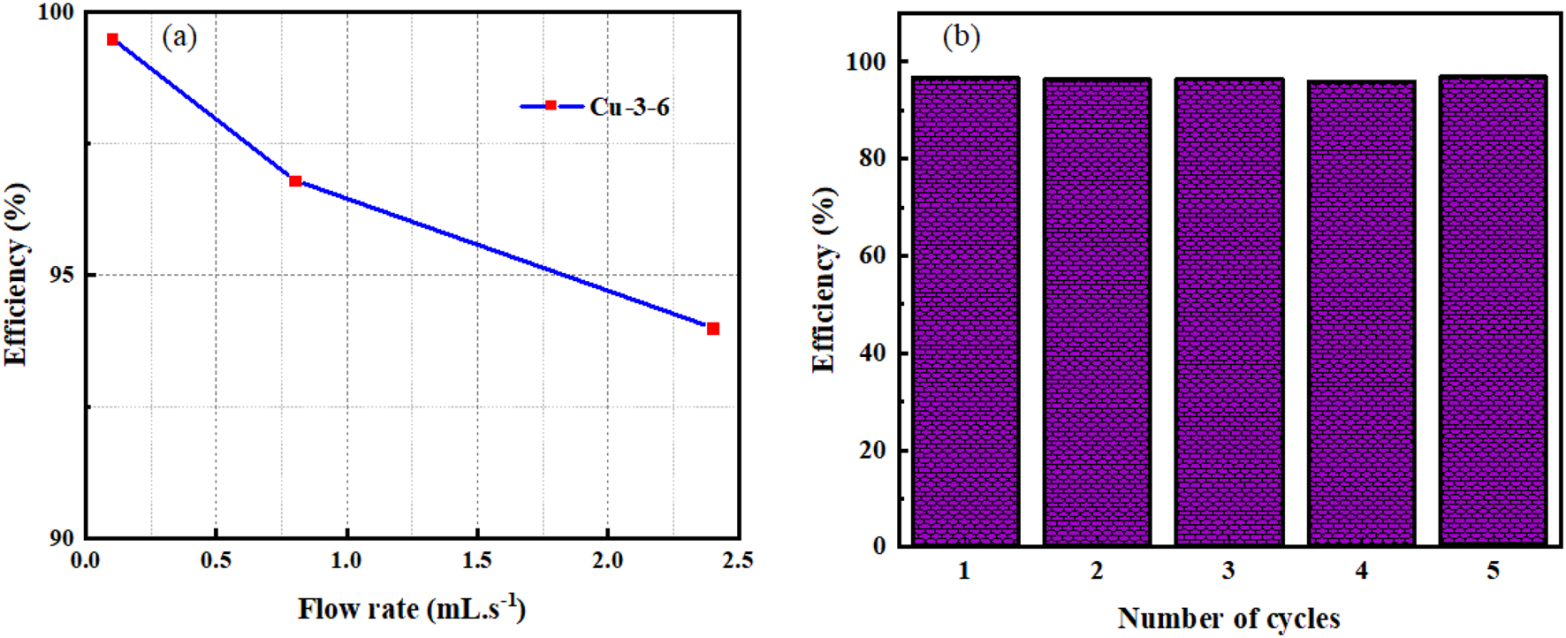
(**a**) Impact of flow rate on oil/water separation efficiency, (**b**) efficiency of oil/water separation in 5 cycles.

The setup shown in Fig. 14 and similar ones, in which water remained on the superhydrophobic mesh, were commonly used in various research^43–46^. Due to covering the mesh surface with water, such setups cannot be generalized in industrial applications, especially for heavy oils’ separation. Therefore, a more scalable setup was tested, Fig. 16. The flow rates differed by adjusting the outlet valve. Oil (dyed with turmeric)/water (dyed with methylene blue) separation efficiency was measured, and the results are shown in Fig. 17a. The efficiency of separation increased by flow rate. The efficiency was 99.5% in a flow rate of 0.1 ml s^−1^. Despite the high efficiency, the low flow rate is not applicable due to the high duration of separation. Therefore, the flow rate of 0.8 ml s^−1^ is optimum for efficiency and separation rate.

The reusability of meshes in this setup was also studied, and efficiency after consecutive recycles with a flow rate of 0.8 ml s^−1^ was obtained (Fig. 17b). Each cycle includes 100 ml of oil and water mixture. The efficiency also showed no considerable decline after five cycles, revealing its reusability.

Table 2 shows a brief aspect of some other research on superhydrophobic mesh fabrication. Comparing the research to this study reveals some superiority of the proposed method. Some benefits are low temperature, low preparation time (16 min), and facility. Efficiency largely depends on the setup designed for experiments and the viscosity of the sample oil. Thus, more than referring to minor differences between various kinds of research is needed.

**Table 2:** Some aspects of other research.

Mesh type	Method	t^1^	T_max_^2^ (°C)	CA (°)	η_O/W_^3^ (%)	References
Stainless steel	(I) Anodizing (II) modification with perfluorooctanoic acid	5 min 48 h	RT^4^	155	96	^1^
Stainless steel	(I) Hydrothermal growth of Ni and Fe double hydroxide structure (II) Modification with polydimethylsiloxane	6 h 0.5 h	120	155	> 99.99	^18^
Stainless steel	(I) Electrodeposition of zinc (II) Solvothermal reaction (III) Modification with SA	25 min 24 h 1 h	140	158	94.54–97.8	^43^
Copper	(I) Cross-machining both sides of the substrate (II) Modification with 1 H, 1 H, 2 H, 2 H-perfluorodecyltriethoxysilane	Negligible 1 h	RT	158.8	98.1	^47^
Copper	(I) Chemical etching (II) Modification with n-dodecanethiol	10 min 20 min	RT	158.7	≥ 96.6	^48^
Stainless steel	(I) Ni/Nip coatings via magnetic field-assisted jet electrodeposition (II) Exposure to air	25 min 6 days	45	154	97.0	^20^
Copper	(I) Chemical oxidation (II) Chemical reaction (III) Modification with 1-dodecanethiol	10 min 20 min 5 min	RT	156 ± 1	96.3	^45^
Copper	(I) Hydrothermal formation of Cu_x_S (II) Modification with SA	6 h 2 h	180	160 ± 1	~ 100	^49^
Brass	(I) Electrochemical etching (II) Modification with SA	6 min 10 min	RT	155.5 ± 3.2	96.8	This study

^1^t: Processing time.

^2^T_max_: Maximum temperature of the treatment process.

^3^η_O/W_: Efficiency of oil/water separation.

^4^RT: Room temperature.

## Conclusion

The superhydrophobic brass mesh with excellent oil/water separation performance was successfully fabricated. The electrochemical etching process was applied for roughening the surface, and SA modification was used to decrease the surface energy of the meshes. The mesh electrochemical etched under 3 V electric potential for 6 min showed larger CA, the highest upward pressure against water drops, and low adhesion. EDS results revealed more zinc dissolution than copper in this sample. This sample was used for oil/water separation treatment in two different setups. The separation efficiency for xylene and ethyl acetate was 95.3 and 95.9%, respectively, in the batch setups. The efficiency of continuous setup increased by decreasing the flow rate and reached 99.5% in the flow rate of 0.1 ml s^−1^. This flow rate is unacceptably slow. Therefore, the flow rate of 0.8 ml s^−1^ with an efficiency of 96.8% was selected as the optimum condition.

## Data Availability

All experimental results have been included in the manuscript. The data can be made available upon reasonable request to the corresponding author.
